# An Initial Low-Dose Etelcalcetide Dosing Strategy in Hemodialysis Patients With Moderate Secondary Hyperparathyroidism is Effective and Cost-Saving

**DOI:** 10.1016/j.ekir.2023.11.009

**Published:** 2023-11-15

**Authors:** Andrea Carta, Martina Tedesco, Federica Mescia, Chiara Manenti, Bernardo Lucca, Federica Verzeletti, Alice Guerini, Federica Gaia Terni, Roberto Zubani, Simona Guerini, Brunella Valzorio, Guido Jeannin, Orsola Carli, Corrado Camerini, Stefano Possenti, Paola Gaggia, Federico Alberici

**Affiliations:** 1 Nephrology Unit, Spedali Civili Hospital, ASST Spedali Civili of Brescia, Brescia, Italy; 2 Department of Medical and Surgical Specialties, Radiological Sciences and Public Health, University of Brescia, Brescia, Italy; 3 Independent Researcher, Brescia, Italy

**Keywords:** calcimimetics, CKD-MBD, etelcalcetide, hemodialysis, PTH, secondary hyperparathyroidism

## Introduction

The intravenous calcimimetic etelcalcetide is an emerging treatment for secondary hyperparathyroidism (SHPT) and chronic kidney disease-mineral and bone disorder (CKD-MBD),^1^ with benefits in terms of reduction of fibroblast growth factor 23 levels and prevention of progression of left ventricular hypertrophy.^2^ However, it remains unclear how best to prescribe this drug in clinical practice. The label recommendation is a starting dose of 5 mg etelcalcetide after hemodialysis, which results in 15 mg/week for patients on a 3 times weekly hemodialysis schedule; the dose is then titrated every 4 weeks according to parathyroid hormone (PTH) and calcium levels. This approach does not take into consideration baseline levels of PTH, and it is possible that a more personalized prescription strategy may be more advantageous. The aim of this work was to explore whether using lower starting doses of etelcalcetide than those recommended by the manufacturer could be a convenient strategy in patients with moderate SHPT.

## Results

This single-center retrospective study included 53 hemodialysis patients with moderate SHPT, defined as PTH between 500 and 1500 pg/ml, who were treated with etelcalcetide for at least 4 months between January 1, 2018 and December 31, 2022. Further details on methods can be found in the Supplementary Methods. Twenty-four patients were started on an etelcalcetide dose ≤7.5 mg/week (“low-dose approach”), whereas 29 patients had an initial dose ≥10 mg/week (“standard approach”). Serum calcium, phosphorus, and PTH were monitored monthly in all patients for dose adjustment.

**Figure 1:**
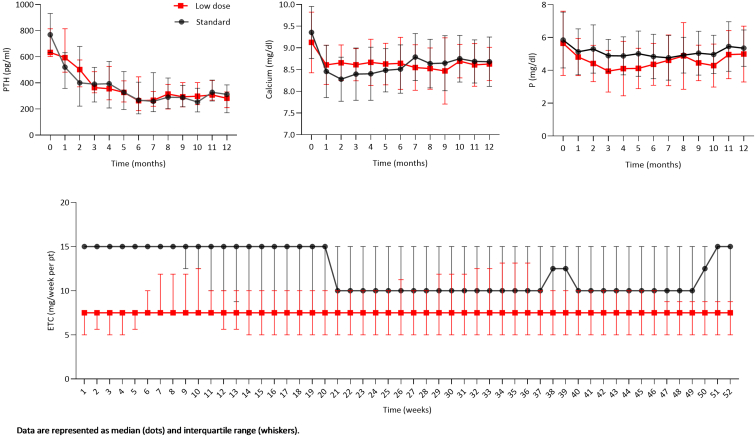
Chronic kidney disease-mineral and bone disorder parameters (parathyroid hormone, calcium and phosphate, assessed monthly) and weekly etelcalcetide dose in the low-dose and standard approaches of etelcalcetide prescription. P, phosphate; ETC, etelcalcetide. Data are represented as median (dots) and interquartile range (whiskers).

Baseline demographics, clinical and CKD-MBD parameters were similar in the 2 groups (Table 1). Median PTH was 633 (601–813) pg/ml in the low-dose group and 769 (630–931) pg/ml in the standard group. Median follow-up was 52 weeks in both groups. Two patients (1 in each group) discontinued etelcalcetide due to excessive PTH suppression and 2 other patients (both in the standard group) stopped the drug because of hypocalcemia or gastrointestinal intolerance (Table 2). During follow-up, CKD-MBD biochemical parameters were comparable between the 2 groups at all time points (Figure 1). At the end of follow-up, PTH levels were 282 (207–332) and 294 (151–382) pg/ml in the low-dose and in the standard group, respectively (*P* = 0.825, Table 2). Most patients (92% in the low-dose group and 90% in the standard group) achieved a decrease in PTH of 30% or greater compared to baseline, and the majority of patients achieved PTH levels within 2 to 9 times the reference levels in both groups at all time points (Supplementary Figure S1).

**Table 1:** Clinical features of 2 groups of patients with different prescription strategies of etelcalcetide, low-dose and standard at baseline, before initiation of the drug

Characteristics	Low-dose (*n* = 24)	Standard (*n* = 29)	*P*-value
Male/female	11/13	17/12	0.353
Age (yrs)	70 (50–78)	63 (52–72)	0.300
Race and ethnicity, *n* (%)			
Asian	3 (13%)	6 (21%)	
Black	2 (8%)	3 (10%)	-
Hispanic	0 (0%)	1 (3%)	
White	19 (79%)	19 (66%)	
Hemodialysis vintage (yrs)	5 (2–8)	5 (3–6)	0.552
Hemodialysis modality			
HD	14	13	0.328
HDF	10	16	
Hemodialysis frequency			
2/wk	2	2	0.844
3/wk	22	27	
Cinacalcet use before switching to etelcalcetide, *n* (%)	3 (9%)	6 (23%)	0.429
Baseline blood tests			
Calcium (mg/dl)	9.0 (8.6–9.6)	9.2 (9.0–9.7)	0.209
Phosphorus (mg/dl)	5.6 (4.4–6.4)	5.5 (4.8–7.4)	0.693
PTH (ng/l)	633 (601–813)	769 (630–931)	0.280
Paricalcitol use (*n*, %)	10 (41%)	7 (24%)	0.174
Initial dose			
Etelcalcetide (mg/w)	7.5 (5–7.5)	15 (15–15)	<0.001
Paracalcitol (mcg/w)	0 (0–10)	0 (0–5)	0.192

HD, hemodialysis, HDF, hemodiafiltration; PTH, parathyroid hormone; wk, weeks; yrs, years.

**Table 2:** Clinical features, drugs doses, duration of follow-up, and cost analysis in low-dose and standard strategy of etelcalcetide prescription during/at the end of follow-up

Characteristics	Low-dose (*n* = 24)	Standard (*n* = 29)	*P*-value
Blood tests at the end of follow-up			
Calcium (mg/dl)	8.7 (8.4–8.8)	8.6 (8.2–9.1)	0.668
Phosphorus (mg/dl)	4.6 (3.7–6.9)	4.7 (4.2–6.2)	0.775
PTH (ng/l)	282 (207–332)	294 (151–382)	0.825
Dose at the end of follow-up			
Etelcalcetide (mg/w)	7.5 (5–7.5)	10 (6.25–15)	0.011
Paracalcitol (mcg/w)	5 (0–10)	5 (0–10)	0.424
Paricalcitol use at the end of follow-up, *n* (%)	17 (71%)	16 (55%)	0.242
Average drug dose/week per patient			
Etelcalcetide (mg/w)	7.6 (6.2–10.2)	10.6 (9.7–15)	<0.001
Paracalcitol (mcg/w)	3.8 (0.2–8.0)	4.4 (1.2–7.9)	0.947
Average drug cost/week per patient			
Etelcalcetide (€/w)	36.6 (29.6–50.0)	50.5 (46.4–71.7)	<0.001
Paracalcitol (€/w)	1.1 (0.0–2.2)	1.5 (0.4–2.2)	0.834
Etelcalcetide + Paracalcitol (€/w)	39.8 (29.8–51.0)	52.9 (47.7–73.8)	<0.001
Cause of etelcalcetide discontinuation, *n* (%)			-
Hypocalcemia	0 (0%)	1 (3%)	
Gastro-intestinal intolerance	0 (0%)	1 (3%)	
PTH over-suppression	1 (4%)	1 (3%)	
Duration of follow-up (weeks)	52 (34–52)	52 (52–52)	0.315

PTH, parathyroid hormone.

Over time, the dose of etelcalcetide remained stable in the low-dose group, whereas in the standard group it tended to decrease, with a trend to rise again over the last weeks of follow-up (Figure 1). Overall, etelcalcetide dose remained consistently higher in the standard group at all the time-points (Figure 1). At the end of follow-up, median weekly etelcalcetide dose was 7.5 (5–7.5) mg in the low-dose and 10.0 (6.25–15) mg in the standard group (*P* = 0.011). The limited sample size precluded the possibility to formally assess whether baseline PTH affected the response. However, when looking at patients with the most marked SHPT in this cohort (PTH >1000 ng/l), there were no obvious differences in PTH response across the 2 etelcalcetide dosing groups (Supplementary Figure S2).

Paricalcitol was prescribed in addition to etelcalcetide in cases with moderate hypocalcemia arising during etelcalcetide treatment (serum calcium <8 mg/dl) and no significant hyperphosphatemia. Use of paricalcitol increased over time in both groups and did not significantly differ according to etelcalcetide dosing strategy (Table 2).

Regarding cost analysis, the median of the average weekly cost of etelcalcetide treatment per patient was €36.6 (29.6–50.0) in the low-dose group and €50.5 (46.4–71.7) in the standard group (*P* < 0.001, Table 2). Considering only patients who completed 1 year of etelcalcetide treatment (*n* = 17 in low-dose and *n* = 23 in standard), the median cost of etelcalcetide per patient-year was €1864 (1332–2713) in the low-dose and €2820 (2486–3728) in the standard group (*P* < 0.001).

## Discussion

In this study, we observed that in a cohort of patients with moderate SHPT, starting etelcalcetide at a lower dose than the one suggested by the manufacturer has similar outcomes to the standard approach in terms of control of CKD-MBD parameters, with a significant reduction in treatment costs. According to data from prospective studies, etelcalcetide dose requirements are lower in patients with less severe SHPT.^3^ Adapting the initial etelcalcetide dose to individual baseline PTH therefore seems a logical approach. The use of a low starting etelcalcetide dose was shown to be a viable alternative in the real-world setting of the Dialysis Outcomes and Practice Patterns Study (DOPPS)^4^: among a cohort of 2596 patients, 27% started at 7.5 mg/week; after 12 months, the pattern of PTH improvement of this subgroup was similar to patients starting at 15 mg/week. However, most patients who started on the low etelcalcetide dose in DOPPS increased the etelcalcetide dose during the 12 months of follow-up. Notably, in our cohort, the median etelcalcetide dose remained stable over the first year in the low-dose group, while partially decreasing initially in the standard group. It is likely that these differences in etelcalcetide requirements reflect different degrees of baseline SHPT in the 2 studies, with our cohort representing a milder spectrum of SHPT than DOPPS. In the latter, despite median baseline PTH levels being similar to our study (762, IQR 510–1158 pg/ml in DOPPS vs. 705, IQR 603–897 pg/ml), there was a markedly higher proportion of patients previously on cinacalcet (49% in DOPPS vs. 17%) and of Black ethnicity (51% in DOPPS vs. 9%), which associates with more severe SHPT.^5^ All these elements support the idea that SHPT was likely more severe in DOPPS than in this study, which included only cases of moderate SHPT by design. We therefore speculate that lower etelcalcetide starting doses are advantageous especially in the setting of less severe SHPT, where our data suggest that this dosing strategy can provide adequate metabolic control over an extended period. Whether these benefits can extend beyond 1 year of treatment will need to be tested in future projects.

Interestingly, we observed treatment-related adverse effects leading to drug discontinuation (namely, hypocalcemia and gastrointestinal intolerance) exclusively in the standard dose group, suggesting that starting with a reduced dose of etelcalcetide may possibly be advantageous from a safety standpoint. However, the limited number of adverse events (2 in total) and the retrospective nature of the study make it impossible to draw any firm conclusion in this respect, which will need to be addressed in adequately powered prospective studies.

A major limitation of this work is the lack of data on fibroblast growth factor 23 and other important endpoints, including cardiac hypertrophy. The equivalence of the low-dose and standard approaches in terms of these important surrogate outcomes should be compared in a prospective fashion. Other limitations are the small sample size and monocentric design. However, the latter also represented a strength, allowing homogeneity in terms of patient monitoring and good data coverage, which is necessary for an accurate cost analysis. Finally, we cannot exclude some residual bias by indication, which is inherent in the retrospective, non-randomized design of this study. Despite the similar clinical profiles of the 2 groups at baseline, with no significant differences in terms of CKD-MBD parameters, it remains possible that the patients felt to have more severe SHPT by the treating physicians were preferentially prescribed the standard dose. This could also account for the trend to increase etelcalcetide dose that was observed in the Standard group during the last weeks of follow-up.

In conclusion, we observed that a low-dose etelcalcetide dosing strategy provided comparable outcomes to the standard approach in hemodialysis patients with moderate SHPT, with significant benefits in terms of costs. Although prospective studies are needed to confirm the advantages of such strategy, close monitoring with regular reassessments remain key to managing etelcalcetide treatment in a safe and cost-effective way, identifying the minimal effective dose for every patient and tailoring prescription to individual clinical evolution.

## Disclosure

FA received consultancy fees from Baxter, AstraZeneca, and Otsuka; is on the advisory board of Trevere Therapeutics, AstraZeneca, and GSK; and received research grant from Amgen. FM received fees for lectures from AstraZeneca, and non-financial support from CSL Vifor. All the other authors declared no competing interests.

## Supplementary Material

Supplementary File (PDF)

**Figure S1.** Percentage of patients with PTH within target levels (2–9 times the reference range) at baseline (month 0) and at months 1 to 12 of follow -up, stratified by etelcalcetide prescription strategy.

**Figure S2.** Longitudinal trends in PTH levels and etelcalcetide dosing in patients with baseline PTH >1000 ng/l receiving either Standard-or Low-dose etelcalcetide.
