# Supplementary material for: An Initial Low-Dose Etelcalcetide Dosing Strategy in Hemodialysis Patients With Moderate Secondary Hyperparathyroidism is Effective and Cost-Saving
Source: Kidney Int Rep. 2023 Nov 15;9(2):482–5. doi: 10.1016/j.ekir.2023.11.009 (PMC10850993; doi:10.1016/j.ekir.2023.11.009)
Supplement: Supplementary File (PDF) [file mmc1.pdf]

Supplementary Methods

This is a single-center, retrospective study carried out at the Nephrology Unit of ASST Spedali Civili di Brescia, Italy, under the ethics of the "Coorte MaRe" project (Ethics number 4945). Data were collected in January 2023 and covered the period from 1 January 2018 to 31 December 2022. The study included patients on chronic HD with a baseline PTH between 500 and 1500 pg/ml who were started on etelcalcetide and received it for at least 4 months during the observation period. The initial etelcalcetide dose was chosen by the prescribing physician according to individual preferences.

Based on the initial dose, patients were retrospectively assigned to either the "Low-dose" (≤7.5 mg etelcalcetide/week) or the "Standard" group (≥10 mg etelcalcetide/week).

Follow-up ended at drug discontinuation, 31 December 2022 or one year since the initial etelcalcetide prescription, whichever earliest.

The two different approaches of etelcalcetide prescription were compared in terms of effects on CKD-MBD related biomarkers and costs. Data were reported as median (interquartile range) or count (percentage), as appropriate. The Mann-Whitney test was used to compare continuous variables between the two groups. Categorical variables were compared using the Chi-square test, with Yate's Continuity Correction in case of expected counts smaller than 5 in at least one cell of the contingency table. Statistical analyses were performed using GraphPad Prism 7 (GraphPad Software, La Jolla, CA, USA).

Supplementary Results

Supplementary Figure 1: Percentage of patients with PTH within target levels (2-9 times the reference range) at baseline (month 0) and at months 1 to 12 of follow-up, stratified by etelcalcetide prescription strategy.

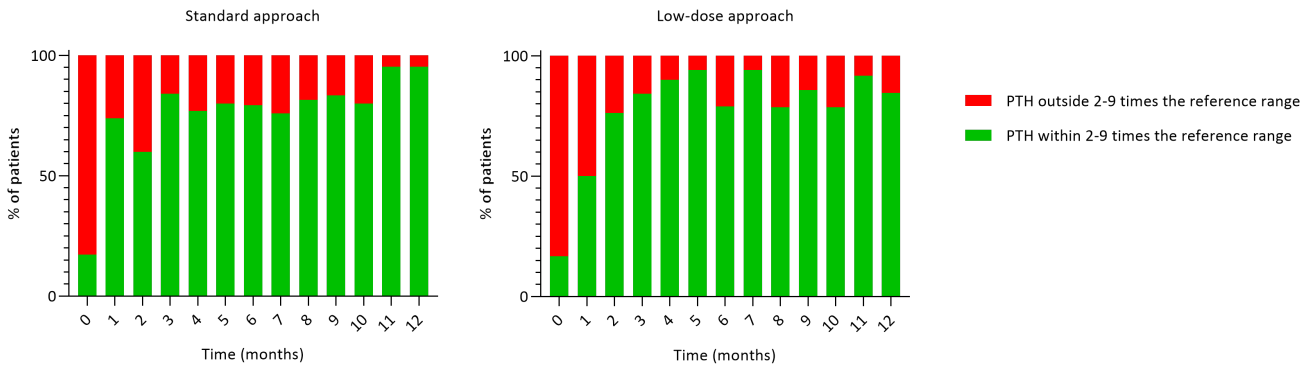

Supplementary Figure 2: Longitudinal trends in PTH levels and etelcalcetide dosing in patients with baseline PTH >1000 ng/l receiving either Standard- or Low-dose etelcalcetide

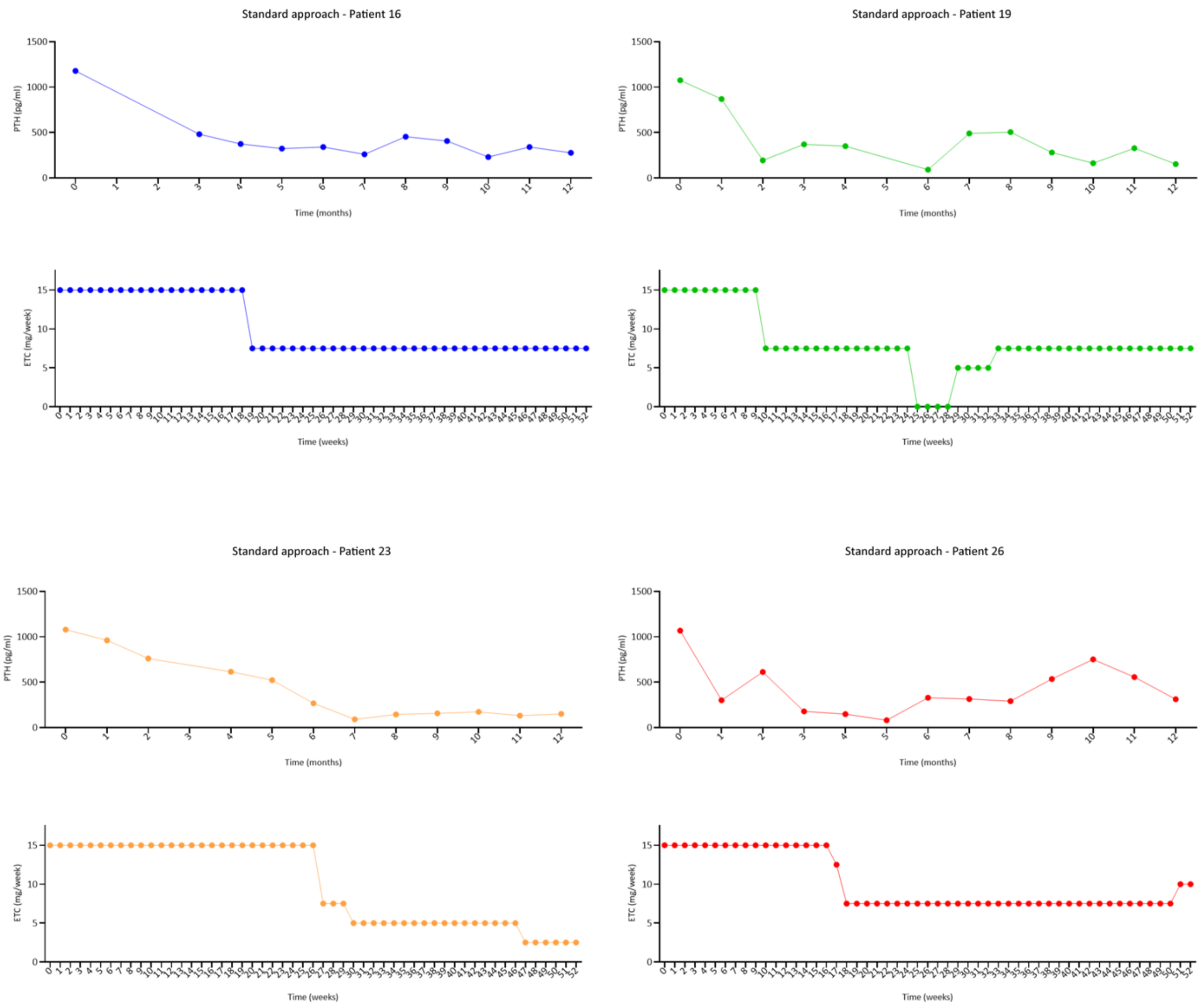

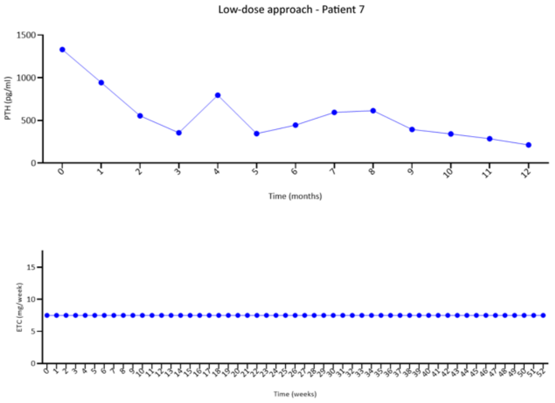
Low-dose approach - Patient 7
PTH (pg/ml)
Time (months)
ETC (mg/week)
Time (weeks)

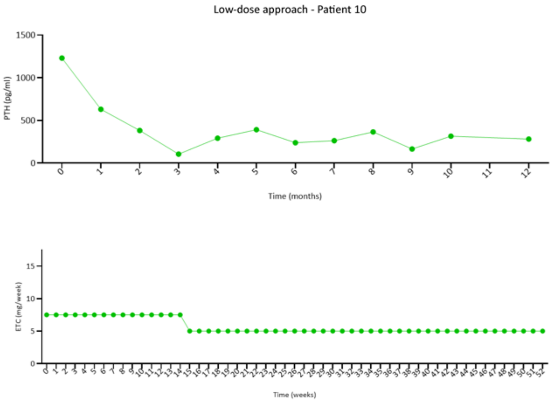
Low-dose approach - Patient 10
PTH (pg/ml)
Time (months)
ETC (mg/week)
Time (weeks)

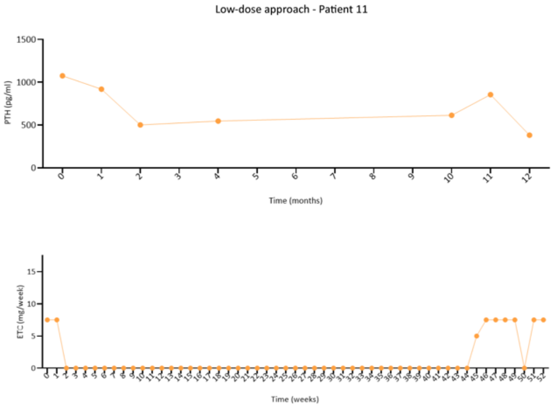
Low-dose approach - Patient 11
PTH (pg/ml)
Time (months)
ETC (mg/week)
Time (weeks)

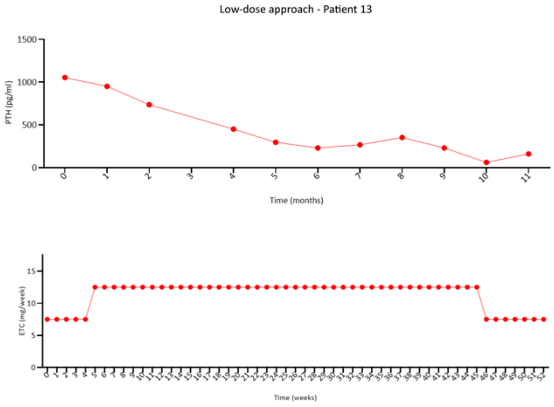
Low-dose approach - Patient 13
PTH (pg/ml)
Time (months)
ETC (mg/week)
Time (weeks)
